# Resilient Stress Reactivity Profiles Predict Mental Health Gains from Online Contemplative Training: A Randomized Clinical Trial

**DOI:** 10.3390/jpm14050493

**Published:** 2024-05-04

**Authors:** Malvika Godara, Tania Singer

**Affiliations:** Social Neuroscience Lab, Max Planck Society, 10557 Berlin, Germany; singer@social.mpg.de

**Keywords:** mindfulness, socio-affective, dyads, mental training, mental health, personalization, plasticity, stress reactivity

## Abstract

Low-dose app-based contemplative interventions for mental health are increasingly popular, but heterogeneity in intervention responses indicates that a personalized approach is needed. We examined whether different longitudinal resilience–vulnerability trajectories, derived over the course of the COVID-19 pandemic, predicted differences in diverse mental health outcomes after mindfulness and socio-emotional dyadic online interventions. The CovSocial project comprised a longitudinal assessment (phase 1) and an open-label efficacy trial (phase 2). A community sample of 253 participants received 12 min daily app-based socio-emotional dyadic or mindfulness-based interventions, with weekly online coaching for 10 weeks. Before and after the intervention, participants completed validated self-report questionnaires assessing mental health. Stress reactivity profiles were derived from seven repeated assessments during the COVID-19 pandemic (January 2020 to March/April 2021) and were categorized into resilient (more plasticity) or vulnerable (less plasticity) stress recovery profiles. After both interventions, only individuals with resilient stress reactivity profiles showed significant improvements in depression symptomatology, trait anxiety, emotion regulation, and stress recovery. Those with vulnerable profiles did not show significant improvements in any outcome. Limitations of this study include the relatively small sample size and potential biases associated with participant dropout. Brief app-based mental interventions may be more beneficial for those with greater levels of stress resiliency and plasticity in response to stressors. More vulnerable individuals might require more intense and personalized intervention formats.

## 1. Introduction

Contemplative interventions, including mindfulness- and compassion-based training, have well-documented benefits for mental health and wellbeing [1,2]. Although traditionally, mindfulness-based contemplative interventions relied on in-person courses supported by teachers, recently, lower-dose web- and app-delivered mindfulness and socio-affective interventions have gained popularity for promoting mental health [3,4]. Meta-analytic evidence supports the effectiveness of online contemplative interventions in reducing depression and anxiety and enhancing resilience [5,6,7,8]. However, despite promising findings, small-to-medium effect sizes indicate heterogeneity in responses to these interventions, suggesting that perhaps some individuals might benefit more than others [9,10,11]. Therefore, efficacy investigations of these interventions are now needed to identify which groups of individuals benefit most from these low-dose online contemplative interventions. Accordingly, recent advances in clinical sciences advocate for a personalized approach to mental health interventions [12].

Thus far, however, most studies have investigated time-stable moderators of intervention responses, such as personality traits, socio-demographic variables, dispositional mindfulness and response styles, or baseline symptom levels [8,10,13]. However, the prevailing models of resilience conceptualize resilient mental wellbeing as a dynamic process of stress recovery that evolves over time in response to encountered life stressors, and not as a mere reflection of specific time-stable psychological aspects [14,15]. Consequently, it can be extrapolated that dynamic stress recovery profiles, derived from an ecologically valid assessment of reactivity to naturalistic stressors, might be more uniquely suited to predicting individual differences in intervention gains since they index the individual capacity for plasticity in response to dynamic contexts, explaining who shows responsivity in a mental training context [16]. To explore this, we examined whether individual variations in adapting to multiple stressors over time could predict differences in mental health benefits from online contemplative interventions. We employed longitudinal resilience and vulnerability profiles, generated over the course of the COVID-19 pandemic and associated lockdowns [17], to predict who would show greater mental health benefits after online mindfulness-based and socio-emotional partner-based interventions [18].

Dynamic stress response profiles have been shown to predict the future state of mental health in prior studies, with more resilient profiles of stress reactivity (i.e., better recovery after stressor, indicating more flexibility in response) being associated with better mental wellbeing at future timepoints as compared to chronic (i.e., poor stress recovery) or delayed dysfunction (i.e., delayed onset of difficulties in stress recovery after stressor) profiles [19]. This indicates that profiles associated with more plasticity in response to stressful situations may be a good indicator of mental wellbeing in the future. Building upon these insights, it is reasonable to anticipate that individuals displaying greater plasticity or more dynamic reactivity in response to naturalistic stressors over extended durations in daily life might also exhibit enhanced plasticity with respect to learning gains during mental interventions. Such a view would align with empirical findings supporting the capitalization view of treatment gains, i.e., those with existing strengths are able to capitalize on them to reap greater benefits from a treatment [20,21]. Accordingly, the compensation versus capitalization model [21] suggests that an intervention could be more effective either (1) for individuals with the greatest difficulties in the areas targeted by the intervention (compensation) or (2) if it builds on the individual’s existing strengths (capitalization). Contrastingly, the compensation approach would suggest that those who show less plasticity over time in response to stressors are those who can profit more from mental training, with empirical support showing that baseline deficiencies predict greater treatment benefits [22,23].

**Figure 1:**
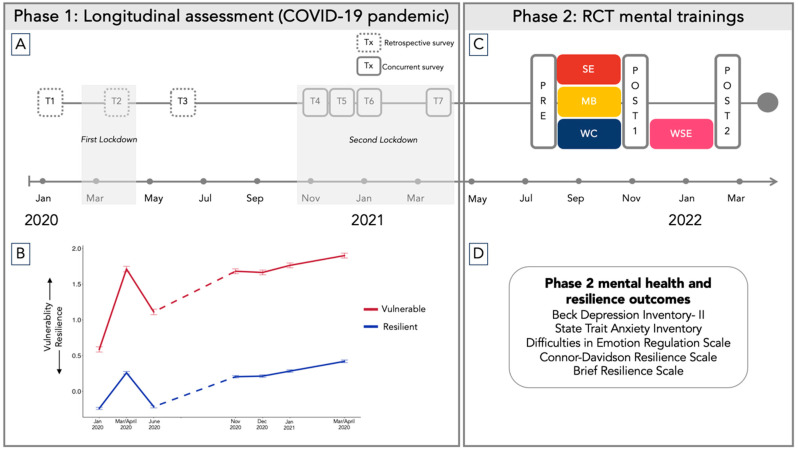
The design of the CovSocial project. (**A**) Phase 1 of the project involving repeated assessment of stress reactivity to various phases of the COVID-19 pandemic in Berlin, Germany. Grey panels indicate phases of state-mandated lockdowns in Germany. Dotted lines indicate retrospective assessment and solid lines represent concurrent assessment. (**B**) The depiction of overall resilience–vulnerability trajectories derived over the seven assessment timepoints in phase 1 of this study. (**C**) The design of the randomized controlled trial (phase 2) conducted with a sub-sample of individuals from phase 1 of this study. SE = socio-emotional intervention group, MB = mindfulness-based intervention group, WC = waitlist control group, WSE = waitlist socio-emotional intervention group, PRE = pre-intervention assessment, POST1 = post-intervention assessment 1, POST2 = post-intervention assessment 2. (**D**) Study measures assessing mental health at pre- and post-intervention stages of phase 2.

In the present study, we explored whether those showing more vulnerable (less plastic) or more resilient (more plastic) dynamic stress recovery profiles [17] showed greater mental health benefits from low-dose online mindfulness-based and socio-affective interventions. Using data from both phases of the CovSocial project [18], the first goal was to investigate whether longitudinal resilience–vulnerability profiles, identified through repeated assessments of stress reactivity during the COVID-19 pandemic in phase 1 [18] (January 2020–March/April 2021; see Figure 1), predicted baseline levels of depressive symptom severity, anxiety vulnerability and symptomatology, emotion regulation (ER) difficulties, and stress recovery and resilience assessed prior to intervention delivery in phase 2 [4]. The second goal of this study was to investigate whether these longitudinal profiles then predicted individual differences in training-related changes in depressive symptoms, anxiety vulnerability and symptomatology, ER difficulties, and resilience after the online socio-emotional or mindfulness-based intervention. The hypotheses for the present study were preregistered on the Open Science Framework as part of the “Mental Health and Resilience” complex of phase 2 of the CovSocial project (osf.io/3nsjc).

## 2. Materials and Methods

### 2.1. Recruitment and Study Design

**Figure 2:**
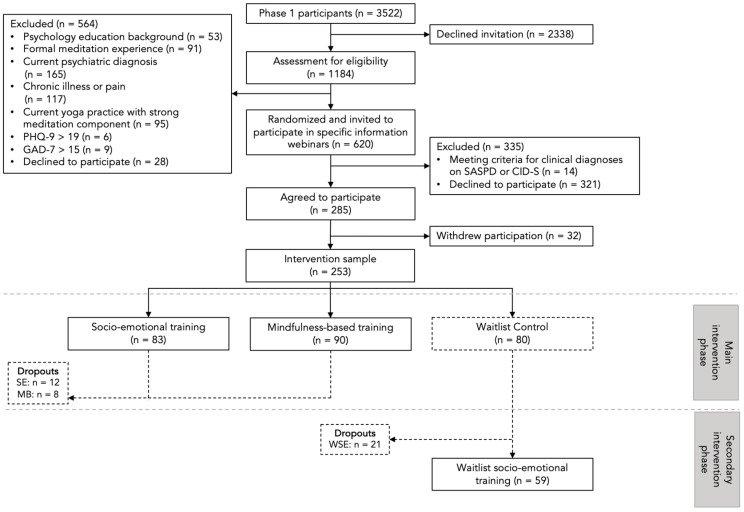
The CONSORT recruitment flow. PHQ-9 = Patient Health Questionnaire-9 [28], GAD-7 = Generalized Anxiety Disorder-7 [29], SASPD = Standardized Assessment of Severity of Personality Disorder [24], CID-S = Composite International Diagnostic—Screener [25], SE = socio-emotional intervention group, MB = mindfulness-based intervention group, WSE = waitlist socio-emotional intervention group. This figure is adapted from a prior study from the CovSocial project [4].

The data for the present study originated from the CovSocial project, which aimed during its initial phase to evaluate shifts in psychological wellbeing amidst the COVID-19 pandemic, and in a subsequent second phase, examined the efficacy of two distinct forms of online mental training (see Figure 1). In phase 1, participants (n = 3522) completed assessments of multiple aspects of mental health, resilience, and social cohesion at seven timepoints: T1 (before lockdown in January 2020), T2 (during first lockdown from mid-March to mid-April 2020), T3 (in June 2020 when restrictions were eased), T4 (November 2020, start of second lockdown), T5 (December 2020), T6 (January 2021), and T7 (mid-March to mid-April 2021, end of second lockdown). In phase 2, as part of a randomized control trial (RCT), a sub-sample of participants from phase 1 (n = 285; see Figure 2 for recruitment flow) were assigned to one of two interventions, partner-based socio-emotional training (SE) or attention-based mindfulness training (MB), or to a waitlist control (WC) group who later underwent socio-emotional training (WSE).

Interested individuals from phase 1 had to meet the following inclusion criteria to take part in phase 2: age between 18 and 65 years, resident of Berlin, access to a smartphone, and proficiency in German language. Participants were pre-screened to exclude vulnerability, educational background in psychology, current or prior meditation practice, experience with stress management programs, chronic illnesses or pain, and history of or current psychiatric diagnosis. Participants were also screened for clinically relevant levels of psychopathology using the Standardized Assessment of Severity of Personality Disorder [24] and Composite International Diagnostic Screener [25].

Power analysis for phase 2 of the project was performed prior to sample recruitment based on biological measures that were part of the phase 2 of the CovSocial project [18]. The a priori effect size was determined and power calculations were performed based on prior work [26], which validated the interventions applied in the present study. Power analyses were conducted using G*Power [27] based on an analysis of variance with repeated measurements and interactions between group and intra-group variables. This comprised the following elements: α = 0.05, power = 0.80, 3 groups, 2 measurements, *r* = 0.39, and *f* = 0.10. The result was a total sample size of n = 297. Therefore, we aimed to recruit around 300 individuals, 100 per intervention group.

We utilized a block randomization technique that was generated by a senior researcher in the project. Participants were randomized in a parallel-group design, with 1:1:1 allocation, using computer-generated numbers. Interventions were assigned to the participants by the study coordinator. The SE and MB groups were tested on the outcome measures at 2 timepoints (pre-test and post-test 1). Meanwhile, the WC group completed the measures at pre-test, post-test 1, and at a third timepoint (post-test 2) after undergoing the intervention. After exclusion and dropouts, 253 participants completed the pre-intervention measures: 83 individuals in the SE group, 90 in the MB group, and 80 in the WC group (sample descriptives in Table 1). For further details, see the study protocol [18]. We invited the first participants to be informed about the interventions in phase 2 of the study on 27 May 2021, and data collection for all phase 2 measures was completed on 31 March 2022. This study was approved by the Ethics Commission of Charité –Universitätsmedizin Berlin (EA4/081/21) and conducted in accordance with the Declaration of Helsinki. All participants provided written informed consent.

### 2.2. Measures

#### 2.2.1. Longitudinal Stress Recovery Profiles

From the phase 1 data, dynamic stress reactivity profiles in response to the COVID-19 pandemic stressors were obtained, which were validated in a multi-step procedure in a prior publication from the project [17]. In the prior study, in the first step, 13 distinct measures of mental health, vulnerability, and resilience (e.g., perceived stress, loneliness, health burden, psychosomatic complaints, life satisfaction, self-efficacy, and coping approaches) were used to extract a latent factor of resilience–vulnerability at each of the seven timepoints of phase 1. A combination of validated scales and self-generated questions was employed, which included the Perceived Stress Scale (PSS-4) for stress perception, the Patient Health Questionnaire-2 (PHQ-2) for depressive symptoms, the Generalized Anxiety Disorder Scale (GAD-2) for anxiety symptoms, and the General Self-Efficacy Short Scale (ASKU) for beliefs about self-efficacy. Additionally, self-generated questions captured pandemic-specific aspects of resilience and vulnerability, such as pandemic-related burdens, psychosomatic complaints, loneliness, stress recovery, coping approaches, optimism, life satisfaction, and the perception of the pandemic as an opportunity. The data were gathered via online surveys conducted repeatedly at seven different time intervals from January 2020 to April 2021. Missing data were addressed using predictive mean matching through the multivariate imputation by chained equations (MICE) method, and a measurement model was specified for each timepoint using confirmatory factor analyses. In the next step, using growth mixture modeling, 4 distinct latent profiles of stress reactivity were identified, which were termed: “most vulnerable”, “more vulnerable”, “more resilient”, and “most resilient”. These profiles were based on longitudinal changes in stress responses to dynamic phases of the COVID-19 pandemic in Germany, such as the first lockdown, re-opening, and second lockdown. The optimal number of classes was determined based on model fit indices and theoretical plausibility. These analyses were conducted using R (version 4.0.3) with the packages mice and lavaan for missing data imputation and measurement model analysis, respectively. Additionally, Mplus (version 8) was employed for growth mixture modeling.

Given the smaller sample size in phase 2, participants in the more and most vulnerable groups were merged into one category termed ‘vulnerable’ (n = 79), and the more and most resilient groups were merged into one ‘resilient’ group (n = 174). Given that we grouped the profiles to ensure adequate statistical power for the present analysis, we tested the mean latent resilience–vulnerability scores for these new ‘vulnerable’ and ‘resilient’ profiles across the 7 timepoints. Within-class comparisons revealed that the vulnerable class did not recover to pre-lockdown levels of vulnerability at re-opening (*p* < 0.001), while in the resilient class, participants recovered to the baseline (*p* > 0.1). When compared to individuals with the resilient profile, individuals with the vulnerable profile had greater levels of vulnerability at the start of the second lockdown (*p* < 0.001), and they showed a steeper increase in vulnerability during the second lockdown (*p* = 0.03). Figure 1B illustrates the resilience–vulnerability time courses of these two groups.

#### 2.2.2. Intervention Outcomes

From the phase 2 data, all intervention outcomes that formed part of the Mental Health and Resilience complex (as outlined in the preregistered strategy on OSF osf.io/3nsjc) were obtained: depressive symptom severity (Beck Depression Inventory-II (BDI-II) [30]), trait anxiety vulnerability and state anxiety symptomatology (State-Trait Anxiety Inventory (STAI-T and STAI-S) [31]), and resilience (Connor Davidson Resilience Scale (CD-RISC [32]) and Brief Resilience Scale (BRS [33])). Moreover, recognizing the significance of emotion regulation (ER) difficulties in mental health [34], we also considered the Difficulties in Emotion Regulation Scale [35] to be a primary outcome in the present study.

### 2.3. Interventions

The SE group, and in the secondary intervention phase the WSE group, engaged in daily 12 min sessions of the Affect Dyad [36], pairing up with a different partner each week, who was randomly assigned to them by the CovSocial mobile app designed for the study. During the Affect Dyad sessions, participants took turns recounting a recent (in the last 24 h) challenging emotional experience and exploring the sensations associated with it in their bodies, followed by sharing a gratitude-inducing moment and reflecting on the bodily sensations evoked by gratitude. The listening partner remained empathetic and non-judgmental but without offering any verbal or non-verbal responses. Meanwhile, participants in the MB group practiced daily 12 min sessions focusing on attention-based mindfulness techniques. Guided by audio meditations, they directed their attention to their breath or to the sounds in their surroundings, or engaged in open awareness meditation, tuning into sensations within themselves and their environment. Both groups were encouraged to practice their respective techniques six times weekly at home, facilitated through the CovSocial mobile app over a 10-week period. Additionally, participants attended weekly two-hour online coaching sessions led by mindfulness and dyad experts, providing a platform with which to discuss and enrich their practice experiences (refer to the supplementary information for the coaching session details). Before the intervention began, every participant underwent a comprehensive 2.5 h introductory session on contemplative training. Additionally, they attended two 2.5 h onboarding webinars designed specifically for the interventions they would receive (refer to the supplementary information “File S1: Mental training protocol for phase 2 of the CovSocial project” for more specifics).

### 2.4. Statistical Analysis

To investigate the first goal, we employed linear models with the stress recovery profile (vulnerable or resilient) as the predictor of pre-test levels of depressive symptom severity, trait anxiety, state anxiety severity, ER difficulties, and resilience (CD-RISC and BRS). To investigate the second goal, we employed separate linear mixed-effects models to assess whether intervention-related changes in each of the outcome measures were predicted by the type of dynamic profile. Each model included a 3-way interaction term between the intervention (SE or MB), time (pre-test or post-test 1), and recovery profile (vulnerable or resilient). A random intercept for the participant was included to account for individual variability in baseline levels of the outcome variables. This allowed for the estimation of individual-specific deviations from the overall group mean, enhancing the accuracy and robustness of the model. Separate models were implemented for the WSE group with a 2-way interaction term between the time of assessment (pre-test, post-test 1 and post-test 2) and recovery profile and a random intercept for the participant. Age and sex were included as covariates in all models, and *p*-values were Bonferroni-adjusted for multiple comparisons. Analyses were conducted in R version 4.3.1 [37] using the *lme4* [38] and *multcomp* [39] packages.

## 3. Results

**Figure 3:**
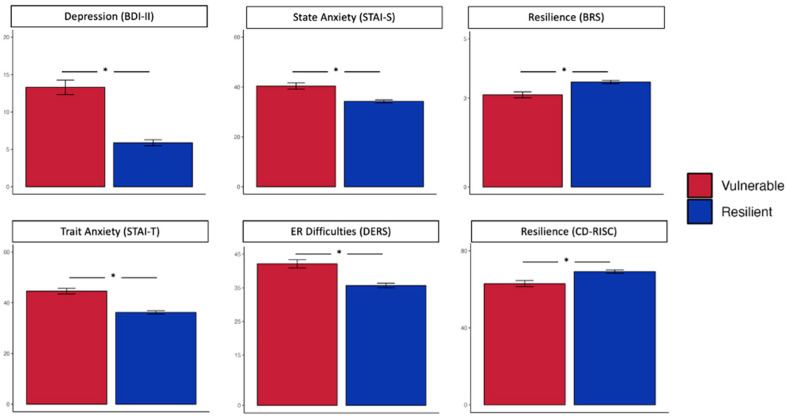
Pre-intervention levels of depressive symptoms, trait anxiety, state anxiety, emotion regulation (ER) difficulties, and resilience (CovSocial project phase 2) stratified by longitudinal vulnerable and resilient response profiles during the COVID-19 pandemic (phase 1). BDI-II = Beck Depression Inventory—II, STAI-T = State Trait Anxiety Inventory—Trait, STAI-S = State Trait Anxiety Inventory—State, DERS = Difficulties in Emotion Regulation Scale, CD-RISC = Connor Davidson Resilience Scale, BRS = Brief Resilience Scale. A significant difference between vulnerable and resilient profiles is indicated by an asterisk (* indicates *p* < 0.05 after adjustment for multiple comparisons).

First, we found that at the pre-intervention stage, individuals displaying the resilient dynamic recovery profile had significantly lower levels of depressive symptoms (*β* = −8.10, *p* < 0.001, d = 1.08), trait anxiety (*β* = −8.57, *p* < 0.001, d = 0.92), state anxiety (*β* = −6.03, *p* < 0.001, d = 0.66), and ER difficulties (*β* = −8.10, *p* < 0.001, d = 1.08) and higher levels of resilience on the CD-RISC (*β* = 6.07, *p* = 0.002, d = 0.48) and BRS (*β* = 0.46, *p* < 0.001, d = 0.58; see Figure 3).

**Figure 4:**
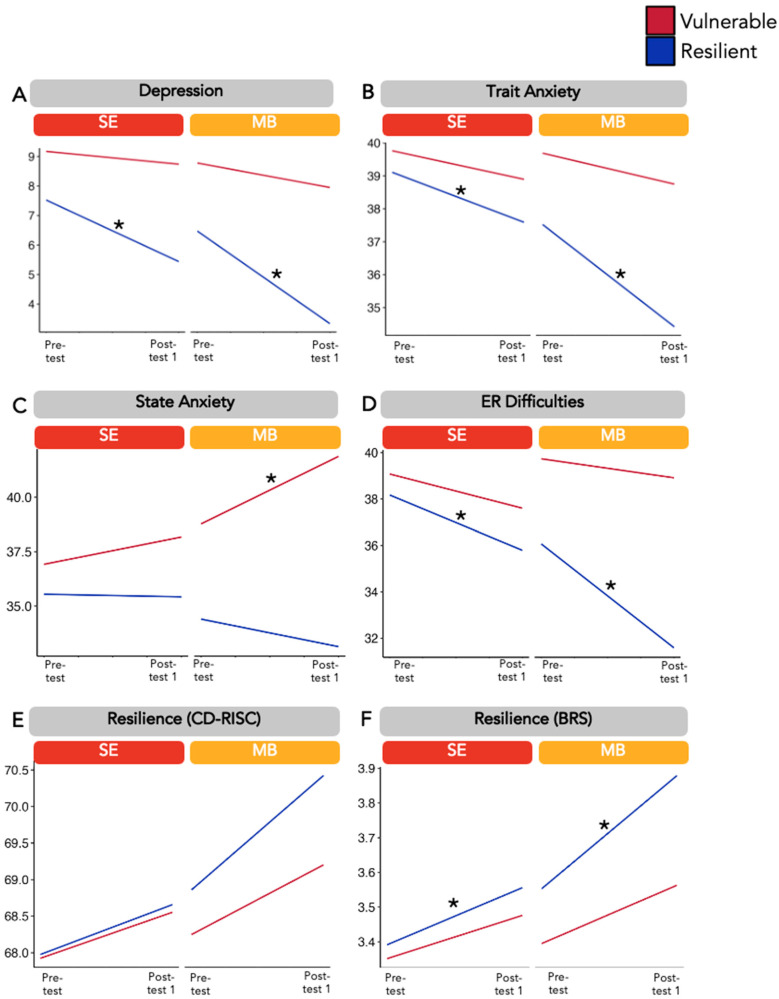
Pre- to post-intervention changes in (**A**) depressive symptoms, (**B**) trait anxiety, (**C**) state anxiety, (**D**) emotion regulation (ER) difficulties, and (**E**,**F**) resilience (CovSocial project phase 2) examined within the context of longitudinal vulnerable and resilient response profiles during the COVID-19 pandemic (phase 1). SE = socio-emotional dyadic intervention, MB = mindfulness-based intervention, CD-RISC = Connor Davidson Resilience Scale, BRS = Brief Resilience Scale. A significant change from pre- to post-intervention is indicated by an asterisk (* indicates *p* < 0.05 after adjustment for multiple comparisons).

Second, mixed-effects models revealed significant three-way (intervention, time, and recovery profile) interactions for depressive symptoms (*F* = 9.37, *p* < 0.001), trait anxiety (*F* = 6.16, *p* < 0.001), state anxiety (*F* = 5.26, *p* < 0.001), ER difficulties (*F* = 10.56, *p* < 0.001), and the BRS (*F* = 7.12, *p* < 0.001), but not for the CD-RISC (*F* = 1.04, *p* = 0.385). The findings are depicted in Figure 4. Post hoc comparisons indicated that the effect of the interventions over time was significantly moderated by the recovery profiles, such that only individuals displaying the resilient recovery profile showed significant decreases in depressive symptomatology in both the SE (*β_SE_* = −2.08, *p* < 0.002, d = 0.34) and MB (*β_MB_* = −3.13, *p* < 0.001, d = 0.51) interventions. Similar findings were observed for trait anxiety (*β_SE_* = −1.52, *p* = 0.044, d = 0.18 and *β_MB_* = −3.11, *p* < 0.001, d = 0.36) and ER difficulties (*β_SE_* = −2.29, *p* = 0.007, d = 0.26 and *β_MB_* = −4.42, *p* < 0.001, d = 0.51). Individuals displaying the vulnerable recovery profile did not show significant changes after either intervention in depressive symptoms (*β_SE_* = −0.43, *p* > 0.5, d = 0.07 and *β_MB_* = −0.82, *p* > 0.5, d = 0.14), trait anxiety (*β_SE_* = −0.87, *p* > 0.5, d = 0.10 and *β_MB_* = −0.94, *p* > 0.5, d = 0.11), or ER difficulties (*β_SE_* = −1.86, *p* = 0.12, d = 0.21 and *β_MB_* = −1.35, *p* = 0.42, d = 0.16). Interestingly, individuals displaying the vulnerable profile showed an increase in state anxiety symptoms in the MB (*β_MB_* = 3.11, *p* = 0.023, d = 0.34) but not the SE (*β_SE_* = 1.25, *p* = 0.53, d = 0.14) intervention. Those with the resilient profile did not show any significant changes in state anxiety in either intervention (*β_SE_* = −0.12, *p* > 0.5, d = 0.01 and *β_MB_* = −1.27, *p* > 0.5, d = 0.14). For changes in resilience on the CD-RISC, we found no significant effect of either stress recovery profile after either the SE (*β_vulnerable_* = 0.63, *p* > 0.5, d = 0.05 and *β_resilient_* = 0.95, *p* > 0.5, d = 0.06) or MB (*β_vulnerable_* = 0.95, *p* > 0.5, d = 0.08 and *β_resilient_* = 1.56, *p* > 0.1, d = 0.13) intervention. Contrastingly, only individuals displaying the resilient profile showed significant increases in stress recovery on the BRS in both interventions (*β_SE_* = 0.17, *p* = 0.017, d = 0.23 and *β_MB_* = 0.33, *p* < 0.001, d = 0.44), which was not the case for individuals with the vulnerable profile (*β_SE_* = 0.13, *p* > 0.1, d = 0.17 and *β_MB_* = 0.17, *p* > 0.1, d = 0.23).

A similar pattern of findings emerged for the underpowered WSE group. We found significant pre- to post-intervention decreases in depressive symptomatology only in the resilient profile (*β_WSE_* = −1.06, *p* = 0.007, d = 0.14) and not in the vulnerable profile (*β_WSE_* = −0.13, *p* > 0.5, d = 0.02). Similar findings were observed for decreases in ER difficulties (*β_vulnerable_* = −1.12, *p* > 0.1, d = 0.12 and *β_resilient_* = −1.10, *p* = 0.02, d = 0.13). On the other hand, there were significant increases in resilience on the BRS only in the resilient profile (*β_WSE_* = 0.11, *p* = 0.02, d = 0.14) and not in the vulnerable profile (*β_WSE_* = −0.03, *p* > 0.5, d = 0.03). There were no significant differences between the two profiles in changes in trait or state anxiety and resilience on the CD-RISC (all *p* > 0.5). Please see the supplementary information’s Figure S1 for a pictorial depiction.

## 4. Discussion

The present study aimed to investigate the predictive power of dynamic longitudinal stress recovery profiles, derived in conditions of naturalistic stressors during the COVID-19 pandemic in Germany, for explaining individual differences in mental health gains after online mindfulness and socio-affective dyadic interventions in the context of the CovSocial project [18]. We explored whether those with more vulnerable or more resilient longitudinal response profiles benefitted more from low-dose online contemplative training programs.

First, we found that individuals displaying more resilient profiles, i.e., those who had lower levels of experienced vulnerability during the COVID-19 pandemic and showed better recovery after stressors (during the period from March 2020 to April 2021), had significantly lower levels of depressive symptoms, trait anxiety, state anxiety, and ER difficulties and higher levels of resilience pre-intervention (in July–August 2021). These findings are in line with prior research that has employed dynamic stress resilience trajectories to predict the future mental health status [19,40].

Second, we found that only individuals displaying resilient profiles showed significant intervention-related decreases in depressive symptomatology, trait anxiety, and ER difficulties and significant improvements in stress recovery after both 10-week online MB and SE interventions. Individuals displaying a vulnerable profile did not show significant improvements after either intervention in any outcome. This indicates that individuals who showed more resilience and plasticity in response to repeated naturalistic stressors during the pandemic were also the ones who benefited more from the online mental training. This aligns with the capitalization view of the compensation versus capitalization model of treatment gains [21]. On the other hand, those showing more vulnerable profiles (less plasticity) during the pandemic did not show significant training-related improvements in mental wellbeing in most measures.

Our findings have crucial theoretical and practical implications. First, the present work adds to the rather limited field of precision contemplative science. Very few prior studies have used data-driven methods, especially dynamic longitudinal profiles, to identify who benefits from app-based contemplative interventions for mental health. A recent study by Webb and colleagues [10,41] employed a machine-learning-based algorithm utilizing baseline characteristics of the individuals, such as baseline levels of distress, depression, and stress. The algorithm identified that those with more baseline levels of distress and psychopathology benefitted more from use of a meditation app. This is in contrast with our findings. While prior studies have typically employed baseline characteristics, or machine learning algorithms based upon baseline characteristics, to predict intervention outcomes, our study diverged by focusing on longitudinal stress recovery profiles. This methodological difference likely accounts for the contrast in findings between our study and previous research. By considering how individuals dynamically respond to varied stressors over a period of time and recover from them longitudinally, our study captures a more nuanced picture of who benefits from contemplative interventions. We investigated the predictive link between long-term adaptability to stressors, evaluated over months, and short-term cognitive plasticity in the context of a 10-week mental intervention, examining how the former impacted the ability to learn and benefit from the latter. Our findings suggest that using data-driven indices that capture this long-term plasticity or dynamic stress recovery process may offer a more comprehensive and accurate prediction of individual differences in intervention responses compared to static baseline characteristics. This underscores the importance of considering dynamic processes in predicting contemplative intervention responses and highlights the potential of precision contemplative science to guide personalized intervention strategies. Building from this, the present findings also hold practical relevance. It can be extrapolated that individuals with more vulnerable response profiles may benefit from more extended and intensified intervention programs, potentially supported by in-person weekly coaching sessions with mindfulness experts, enabling them to fully experience the mental health benefits of the online contemplative interventions utilized in this study.

Interestingly, we found that individuals displaying vulnerable profiles showed an increase in state anxiety after the MB but not the SE intervention. This finding supports the Monitor and Acceptance Theory, which suggests that mindfulness-based interventions that enhance the monitoring of bodily sensations without including acceptance components can, in fact, lead to a worsening of anxiety symptomatology [42]. Supporting this view, the SE intervention, which incorporates both monitoring and acceptance aspects of contemplative interventions [36], did not lead to significant increases in anxiety symptomatology for the vulnerable group, indicating a buffering effect.

## 5. Limitations

Some limitations of the present work must be considered. Foremost, due to the longitudinal design of this study, the final sample size of individuals displaying vulnerable profiles was rather small. Therefore, it is possible that the vulnerable group was not sufficiently powered for us to detect significant effects. This limitation could have contributed to the null findings observed in certain analyses, suggesting the need for caution when interpreting these subgroup effects. Furthermore, we observed participant dropouts over the course of phase 2 of the study, particularly between study onboarding and the pre-intervention assessment phase, constituting approximately 11.2% of the initial sample (32 out of 285 participants). There was also specific attrition in the waitlist control group between the pre-intervention and post-intervention 1. This dropout phenomenon reflects the challenges inherent in longitudinal research involving contemplative interventions, such as the one implemented here, which required daily practice and weekly 2 h coaching sessions. However, to address this issue and ensure the integrity of the randomized design, an intention-to-treat approach was employed, which involves analyzing participants according to their original assigned group, regardless of dropout. This provides a more conservative estimate of intervention effects and accounts for potential biases introduced by dropout. Moreover, participants from phase 1 who further volunteered to participate in the intervention study of phase 2 may have been more motivated or interested in contemplative interventions than the general population, leading to self-selection bias. Although we tried to control this aspect of the self-selection bias through our use of a randomized controlled study design and the inclusion of demographic variables as covariates in our statistical analyses, self-selection bias cannot be ruled out. However, it is important to acknowledge that in contrast to most mental training studies involving mindfulness-based interventions, here, participants were initially recruited for a large-scale COVID-19-related mental health study based on random draws of addresses from the Berlin city register, rather than specifically for an intervention study. Additionally, this study’s longitudinal design in phase 1 and rather heterogeneous sample may have helped to capture a broad range of perspectives and experiences, thereby partially addressing potential self-selection biases. Note, as well, that we excluded any person with prior experiences with any sort of contemplative interventions or practices. Future studies could benefit, however, from larger sample sizes, more heterogenous samples, more objective measures, and additional follow-up periods. Future research should also explore whether brief in-person interventions and more person-tailored training approaches are needed in vulnerable populations. Additionally, an intentionally wide age range of participants was recruited for this study, to enhance the generalizability of our findings across different age groups. However, this approach may have introduced age-related heterogeneity within the sample, potentially influencing the results. While our study considered age and gender as covariates, their inclusion did not yield significant findings, suggesting that age or sex differences may not have substantially impacted our findings. However, it is plausible that other socio-demographic factors or individual characteristics, such as socio-economic status, educational level, or cultural background, could have influenced the intervention outcomes but were not explored in the current study. Thus, future research could benefit from a more comprehensive examination of these factors, to further our understanding of their roles in shaping the effectiveness of personalized mental health interventions. Additionally, investigating other potential moderators, such as personality traits or coping styles, may provide further insights into how individual differences impact intervention responses. Moreover, exploring the interplay between socio-demographic factors and intervention outcomes could inform the development of more tailored and culturally sensitive interventions, to address mental health needs effectively across diverse populations.

## 6. Conclusions

Our study’s strength lies in the employment of data-driven longitudinal stress response profiles, indexing dynamic stress recovery in response to the COVID-19 pandemic, to predict who reaps more mental health benefits from low-dose online contemplative interventions in a community sample. Our findings highlight the importance of targeting interventions to specific stress recovery profiles, as the effectiveness of the interventions could vary depending on the individual’s plasticity profile. In line with the Precision Medicine Initiative^®^ led by the National Institute of Health (NIH) and the National Institute of Mental Health (NIMH) Strategic Plan [43], this suggests that a one-size-fits-all approach to mental health and app-based contemplative interventions may not be effective. These findings suggest that interventions targeted towards individuals with a more vulnerable stress recovery profile may need to be tailored differently from those for individuals with a more resilient profile. Individuals with a vulnerable profile may require more intensive, longer-term, or in-person interventions, whereas those with a resilient profile can benefit from a relatively low-dose 10-week online intervention.

## Figures and Tables

**Table 1:** An overview of the intervention sample (n = 253). This table is adapted from a prior study from the CovSocial project [4]. SE = socio-emotional, MB = mindfulness-based, WC = waitlist control.

Characteristic	SE	MB	WC
N	83	90	80
Mean age (SD)	43.14 (11.80)	44.14 (11.44)	45.86 (11.15)
Female participants, N (%)	65 (78.3%)	64 (71.1%)	62 (77.5%)
Migration background (to current country of residence), N (%)	4 (4.8%)	10 (11.1%)	3 (3.8%)
Years of education, mean (SD)	18.49 (3.97)	17.06 (3.52)	18.41 (3.21)
Married or cohabiting, N (%)	27 (32.5%)	32 (35.6%)	32 (40%)
Lifetime prevalence of psychiatric disorder	17 (21.0%)	16 (17.8%)	18 (22.5%)
Income > Berlin average monthly net income (EUR 2175 (as reported by the Department of Statistics of Berlin-Brandenburg (2019)))	52 (62.7%)	61 (67.8%)	56 (70.9%)
Full-time employment, N (%)	42 (50.6%)	57 (63.3%)	46 (57.5%)

## Data Availability

Data will be made available upon request.

## Supplementary Materials

The following supporting information can be downloaded at https://www.mdpi.com/article/10.3390/jpm14050493/s1. File S1: Mental training protocol for phase 2 of the CovSocial project. Figure S1: Post-test 1 to post-test 2 changes in intervention outcomes in WSE group stratified by longitudinal stress reactivity profiles.
