# Supplementary figures and images for: Resilient Stress Reactivity Profiles Predict Mental Health Gains from Online Contemplative Training: A Randomized Clinical Trial
Source: J Pers Med. 2024 May 4;14(5):493. doi: 10.3390/jpm14050493 (PMC11121773; doi:10.3390/jpm14050493)

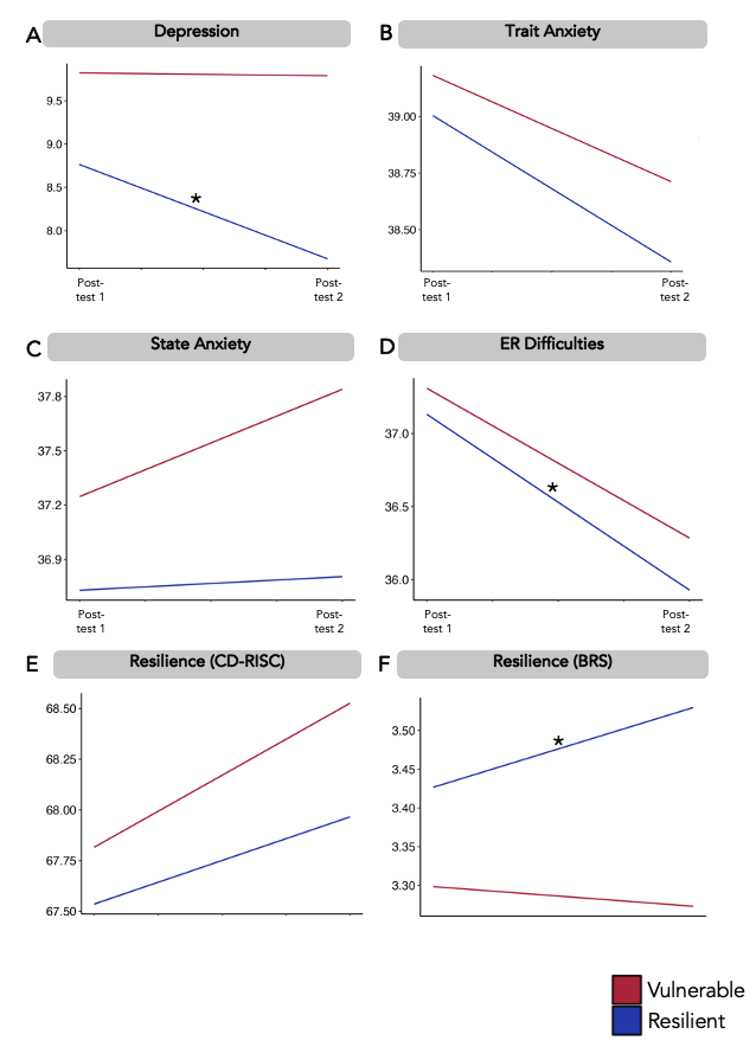

Supplement: Supplementary file 1 [file jpm-14-00493-s001.zip › Figure S1 Post-test 1 to post-test 2 changes in intervention outcomes in WSE group stratified by longitudinal stress reactivity profiles..png]
